# A canine-specific anti-nerve growth factor antibody alleviates pain and improves mobility and function in dogs with degenerative joint disease-associated pain

**DOI:** 10.1186/s12917-015-0413-x

**Published:** 2015-04-30

**Authors:** B Duncan X Lascelles, David Knazovicky, Beth Case, Mila Freire, John F Innes, Alexander C Drew, David P Gearing

**Affiliations:** Comparative Pain Research Laboratory, Department of Clinical Sciences, College of Veterinary Medicine, North Carolina State University, Raleigh, NC USA; Center for Comparative Medicine and Translational Research, College of Veterinary Medicine, North Carolina State University, Raleigh, NC USA; Center for Pain Research and Innovation, UNC School of Dentistry, Chapel Hill, NC USA; Faculté de médecine vétérinaire, Université de Montréal, Saint-Hyacinthe, QC Canada; ChesterGates Referral Hospital, Chester, UK; Nexvet Australia Pty Ltd, Melbourne, VIC Australia

**Keywords:** Nerve growth factor, Dog, Antibody, Pain, Model, Osteoarthritis, Accelerometry, Actimetry

## Abstract

**Background:**

There is a critical need for proven drugs other than non-steroidal anti-inflammatory drugs for treatment of degenerative joint disease (DJD) pain in dogs. Antibodies against nerve growth factor (NGF) are analgesic in rodent models and in humans with DJD. This pilot study aimed to evaluate the efficacy of a novel caninised anti-NGF antibody (NV-01) for the treatment of DJD pain in dogs. In a randomized, parallel group, stratified, double masked, placebo controlled, proof of principle clinical pilot study design, 26 dogs with DJD received NV-01 (200 mcg/kg IV) or placebo on day 0 (D0). In addition to objective accelerometry measures, owners completed clinical metrology instruments (Client-Specific Outcome Measures [CSOM], Canine Brief Pain Inventory [CBPI] and Liverpool Osteoarthritis in Dogs Index [LOAD]) on D0, D14 and D28. CBPI subscales (pain severity [PS] and pain interference [PI]), CSOM and LOAD scores were evaluated within and between groups for change over time. Recognized success/failure criteria were applied and success compared between groups.

**Results:**

CBPI PS and PI scores significantly improved in the NV-01 group (PS: D0-14, P = 0.012 and D0-28, P = 0.019; PI: D0-14, P = 0.012 and D0-28, P = 0.032) but not in the placebo group. CSOM scores showed similar patterns with a significant difference between within-group changes at D14 and D28 (P = 0.038 and P = 0.009, respectively), and significantly more successes at D28 (P = 0.047). LOAD scores significantly improved in the NV-01 group (D0-14, P = 0.004 and D0-28, P = 0.002) but not in the placebo group. There were significant differences between the groups for change in LOAD score at D14 (P = 0.014) and D28 (P = 0.033). No side effects were noted. Activity in the NV-01 group increased over the study period compared to placebo (P = 0.063) and the difference between the groups for change in activity over the time period 9am-5pm (8 hours) was significant (P = 0.006).

**Conclusions:**

These pilot data demonstrate a positive analgesic effect of anti-NGF antibody in dogs suffering from chronic pain. The magnitude of the effect appeared identical to that expected with an NSAID.

**Electronic supplementary material:**

The online version of this article (doi:10.1186/s12917-015-0413-x) contains supplementary material, which is available to authorized users.

## Background

In veterinary medicine, the mainstay of drug therapy for the alleviation of clinical signs associated with osteoarthritis (OA) or degenerative joint disease (DJD)-associated pain in dogs are the non-steroidal anti-inflammatory drugs (NSAIDs). This may be partly due to the fact that there are no other classes of drug approved by the Food and Drug Administration – Center for Veterinary Medicine (FDA-CVM) for the control of DJD-associated pain in dogs. Despite some data to indicate efficacy of other drugs for OA-associated pain in dogs [1], evidence-based data indicates NSAIDs are currently considered the most effective therapy for DJD-associated pain [2-4]. However, NSAIDs are not always sufficiently effective [1] and concerns about side effects result in a large unmet need in the treatment of canine DJD-associated pain.

Targeting nerve growth factor (NGF) has emerged as a potentially useful therapeutic avenue for pain control. NGF was originally identified as a critical factor for the development and maintenance of sensory and sympathetic neurons in the developing nervous system. However, it is now clear that the dependence of these neurons on NGF for survival is restricted to a brief time during development and in the adult system, NGF has an important role in pro-nociception via its effects on the NGF-specific tyrosine kinase receptor (TrkA) (reviewed in [5]).

NGF binds to the high-affinity NGF-specific TrkA which results in autophosphorylation of the TrkA intracellular domain and activation of subsequent downstream signaling cascades (reviewed in [5]). This results in post-translational changes in the transient receptor potential vanilloid receptor 1 (TRPV1) cation channel, increasing its excitability, and further upregulation of other proteins that increase the excitability of the primary afferent fiber [5]. NGF also activates mast cells, and thus further sensitizes neurons as a result of the products released by mast cells [6]. Given its role in nociception, various ways of preventing activation of TrkA have been explored, including removing free NGF, preventing NGF binding to TrkA or preventing activation of TrkA [7]. Of these approaches, neutralizing monoclonal antibodies (mAb) targeting NGF (‘removing free NGF’) have been developed first.

Inhibition of NGF function via anti-NGF antibodies markedly reduces hyperalgesia and behavioral indicators of pain in various animal models of inflammatory arthritis [8,9]. In human clinical studies, several anti-NGF mAbs have been evaluated and been shown to reduce pain and improve function in patients with OA [10-14].

However, it is apparent that pain disorders display significant differences in their responsiveness to anti-NGF agents [15].

Recently, a canine-specific mAb against NGF (termed NV-01) was described and reported to have high affinity and potency, no effector activity, a long half-life and low immunogenic potential [16]. One recent report suggested that this caninised anti-NGF mAb may provide alleviation of the signs of pain in dogs with osteoarthritis [17]. In that study, all dogs received the mAb with the time of administration being randomized and blinded. Although this innovative design provides initial information on the efficacy of this NGF-neutralizing antibody, the pervasive placebo effect known to occur in canine OA trials [18] and the lack of objective measures used indicated that a more comprehensive study design was desirable to fully assess the potential utility of this therapeutic [17].

The aim of the present study was to further assess the pain alleviating and function enhancing effects of NV-01 in dogs with DJD-associated pain and mobility impairment. Our hypothesis was that a single treatment of NV-01 would decrease pain and improve mobility in dogs with DJD-associated pain for 4 weeks, as measured by owner-completed clinical metrology instruments (CMIs) and objectively measured activity. Specifically, using a randomized, parallel group, stratified, double masked, placebo controlled, proof of principle clinical pilot study design we aimed to assess the pain-alleviating and activity enhancing effects of NV-01 in dogs with DJD-associated pain, using the primary outcome measures of CMIs and actimetry data. In addition, we aimed to evaluate the effects of NV-01 on index joint pain, total joint pain score, owner-assessed side effects, hematology, clinical chemistry and urinalysis as secondary outcome measures.

## Results

**Figure 1:**
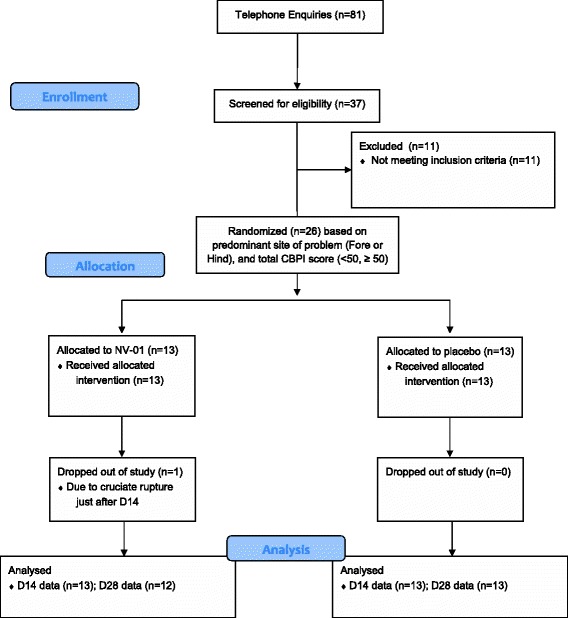
Study participant flow diagram.

The study was conducted between May and December 2013. Eighty-one enquiries from interested dog owners were received and following a telephone discussion, this resulted in 37 screening appointments (Figure 1). Of these, there were 11 screening failures: 4 for lack of radiographic findings of DJD; 3 for insufficient degree of impairment on the CSOM (Client-Specific Outcome Measures); 2 because of significant stifle instability associated with cruciate ligament rupture; 2 due to concurrent neurologic disease. Twenty-six dogs entered the study. There were no differences between the groups with respect to age, sex distribution, body weight or body condition score (Table 1). There was even distribution of index joints/regions across the two groups (Table 2).

**Table 1:** **Enrolled dog demographic summary on D0 of study (treatment day)**

	**NV-01**	**Placebo**	**P-value for comparison of treatment groups**
Age, years	10 (4-14)	10 (4-16)	0.795 (Wilcoxon Rank Sums)
Sex	8FS; 4MC; 1M	5FS; 7MC; 1M	0.470 (Pearson)
Weight, kilograms	27.0 (16.3-40.5)	30.2 (19.9-44.8)	0.209 (ChiSq)
Body condition score (median)	5 (5-8)	6 (5-7)	0.713 (Wilcoxon Rank Sums)

**Table 2:** **Index joint or index axial skeleton region distribution of the enrolled dogs on D0**

**Index joint/region**	**NV-01 group**	**Placebo group**
Elbow	3	3
Stifle	1	1
Hip	5	7
Neck	0	1
Lumbar	1	0
Lumbo-Sacral	3	1

Of the 26 dogs, 25 completed the 28-day study (Figure 1). One dog was withdrawn just after the D14 assessment due to cruciate ligament rupture. This occurrence was not considered to be treatment related.

### Canine brief pain inventory pain severity score (CBPI PSS)

The treatment group improved significantly over time (at D14, P = 0.012 and at D28, P = 0.019). The placebo group did not improve significantly over time (at D14, P = 0.164 and at D28, P = 0.347). There were no statistical differences between the groups at any time point for absolute scores, or change in scores. These scores and changes are tabulated in Table 3. Using a comparison of changes in CBPI PSS scores, the treatment effect size was 0.56 at D14 and 0.46 at D28.

**Table 3:** **Summary of medians, range and statistical comparisons for the Canine Brief Pain Inventory Pain Severity Scores (CBPI PSS) at D0, D14 and D28 of the study**

**Group**	**D0**	**D14**	**D28**	**D0 minus D14**	**Within group change P-value**	**D0 minus D28**	**Within group change P-value**
NV-01	4.75 (1.75-9.00)	2.00 (0.50-6.25)	2.63 (1.00 – 7.00)	1.50 (-0.75 – 4.50)	**0.012**	1.50 (-0.75 – 4.00)	**0.019**
Placebo	3.75 (1.25-8.50)	3.00 (1.00-8.75)	3.00 (1.00 – 8.00)	0.75 (-2.50 – 4.25)	0.164	0.75 (-2.00 – 4.25)	0.347
Between group comparison P-value	0.898	0.719	0.744	0.226		0.210	

Decreases in scores signify improvement, and positive values for change indicate improvement. Significant P-values are denoted in bold type.

### Canine brief pain inventory pain interference score (CBPI PIS)

The treatment group improved significantly over time (at D14, P = 0.012 and at D28, P = 0.032). The placebo group did not improve significantly over time (at D14, P = 0.06 and at D28, P = 0.11). There were no statistical differences between the groups at any time point for absolute scores, or change in scores. These scores and changes are tabulated in Table 4. Using a comparison of changes in CBPI PIS scores, the treatment effect size was 0.73 at D14 and 0.38 at D28.

**Table 4:** **Summary of medians, range and statistical comparisons for the Canine Brief Pain Inventory Pain Interference Scores (CBPI PIS) at D0, D14 and D28 of the study**

**Group**	**D0**	**D14**	**D28**	**D0 minus D14**	**Within group change P-value**	**D0 minus D28**	**Within group change P-value**
NV-01	4.50 (1.17 – 9.00)	2.20 (0.20 – 6.30)	3.17 (0.67 – 8.17)	1.63 (-0.63 – 5.37)	**0.012**	1.92 (-2.50 – 4.34)	**0.032**
Placebo	4.67 (1.83 – 9.00)	3.00 (0.80 – 8.50)	3.17 (1.33 – 7.50)	1.03 (-2.17 – 3.20)	0.06	0.5 (-1.00 – 3.83)	0.110
Between group comparison P-value	0.939	0.106	0.384	0.0734		0.289	

Decreases in scores signify improvement, and positive values for change indicate improvement. Significant P-values are denoted in bold type.

### Canine brief pain inventory success/failure

At D14, there were more successes in the NV-01 group (6/12, 50%) than the placebo group (1/12, 8.3%) (P = 0.069). Again, at D28 there was no difference between groups in success/failure (P = 0.214) but there were more successes in the NV-01 group (6/11, 55%) than the placebo group (3/12, 25%).

### Client specific outcome measure (CSOM)

The NV-01 group improved significantly over time (at D14, P = 0.004 and at D28, P = 0.001). The placebo group improved significantly over time at D14, P = 0.031, but not at D28, P = 0.078. At D14 and D28, the NV-01 group had lower scores (better ability to perform the activities) than the placebo group (P = 0.011 and P = 0.032 respectively). Additionally, the degree of improvement over D0 to D14, and D0 to D28 was significantly greater in the NV-01 group than the placebo group (P = 0.038 and P = 0.009 respectively). These scores and changes are tabulated in Table 5.

**Table 5:** **Summary of medians, range and statistical comparisons for the Client Specific Outcome Measures scores (CSOM) at D0, D14 and D28 of the study**

**Group**	**D0**	**D14**	**D28**	**D0 minus D14**	**Within group change P-value**	**D0 minus D28**	**Within group change P-value**
NV-01	6.0 (4 – 11)	4.0 (0 – 7)	3.5 (0 – 9)	3.0 (-1 – 8)	**0.004**	3.0 (0 – 6)	**0.001**
Placebo	6.0 (5 – 11)	5.0 (3 – 8)	6.0 (3 – 9)	0.0 (-1 – 3)	**0.031**	0.0 (-1 – 3)	0.078
Between group comparison P-value	0.548	**0.011**	**0.032**	**0.038**		**0.009**	

Decreases in scores signify improvement, and positive values for change indicate improvement. Significant P-values are denoted in bold type.

At D14 there were more successes in the NV-01 group (9/13, 69%) versus the placebo group (6/13, 46%), but this was not significant (P = 0.269). At D28, there was a significant difference between groups in the number of successes (P = 0.047) with 8/12 (67%) being classified as successes in treatment group versus 3/13 (23%) in the placebo group.

### Liverpool osteoarthritis in dogs (LOAD)

**Figure 2:**
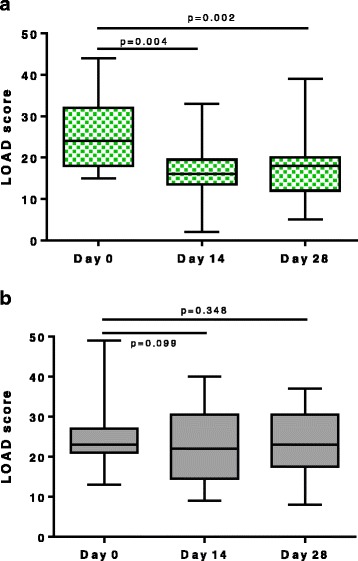
Change in LOAD scores over time in dogs administered anti-NGF antibody or placebo. **a**. Change from baseline in LOAD (median [solid line], interquartile range [shaded area] and extreme values [whiskers]) in dogs treated with a single 200 mcg/kg intravenous injection of anti-NGF antibody on D0. **b**. Change from baseline in LOAD (median [solid line], interquartile range [shaded area] and extreme values [whiskers]) in dogs treated with a single intravenous injection of placebo (saline) on D0.

The NV-01 group improved significantly over time (at D14, P = 0.004 and at D28, P = 0.002) (Figure 2a). The placebo group did not improve over time (at D14, P = 0.099, and at D28, P = 0.348) (Figure 2b). There were no significant differences between the groups at D0, D14 or D28 in total LOAD scores. However, the change in score over D0 to D14, and D0 to D28 was significantly greater in the NV-01 group than the placebo group (P = 0.014 and P = 0.033 respectively). These scores and changes are tabulated in Table 6. Using a comparison of changes in LOAD scores, the treatment effect size was 1.15 at D14 and 0.96 at D28.

**Table 6:** **Summary of medians, range and statistical comparisons for the Liverpool Osteoarthritis in Dogs Index (LOAD) at D0, D14 and D28 of the study**

**Group**	**D0**	**D14**	**D28**	**D0 minus D14**	**Within group change P-value**	**D0 minus D28**	**Within group change P-value**
NV-01	24.0 (15-44)	16.0 (2-33)	18.0 (5-39)	11.0 (-3-19)	**P = 0.004**	5.5 (-2-18)	**P = 0.002**
Placebo	23.0 (13-49)	22.0 (9-40)	23.0 (8-37)	2.0 (-6-10)	P = 0.099	1.0 (-7-12)	P = 0.348
Between group comparison P-value	P = 1.00	P = 0.149	P = 0.200	**P = 0.014**		**P = 0.033**	

Decreases in scores signify improvement, and positive values for change indicate improvement. Significant P-values are denoted in bold type.

### Activity monitoring

**Figure 3:**
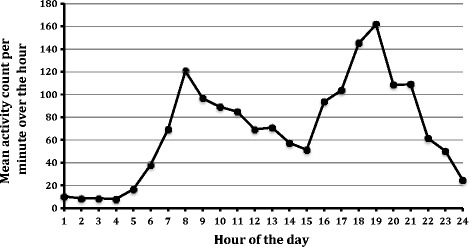
Graph of the mean activity count per minute over each hour of the day for all dogs during the baseline period.

**Figure 4:**
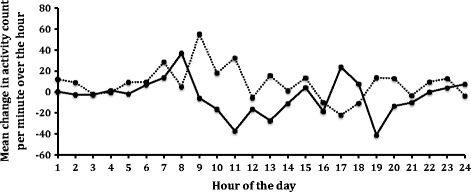
Graph of mean change (week 4 minus baseline) in activity count per minute for each hour of the day in the NV-01 group (dotted line) and the placebo group (solid line).

The mean activity over 24 hours during the baseline period (Days -7 to -1) is shown in Figure 3. Using the average activity per minute over the baseline and final week (Days 20-27), activity in the NV-01 group increased over the duration of the study (significant on 1-sided *t*-test, P = 0.045; significant at the 10% level on 2-sided *t*-test, P = 0.090). Activity in dogs in the placebo group did not change over the duration of the study (1-sided *t*-test, P = 0.810; 2-sided *t*-test, P = 0.379). The difference between the groups for change in average activity was significant at the 10% level (P = 0.063, 2-sided *t*-test), but not at the critical p-value (0.05) set *a priori* before the study initiated. Figure 4 illustrates the change (final week minus baseline) in mean activity per minute over 24 hours. The difference between the groups for change in activity over the time period 9am-5pm (8 hours) was significant, with the NV-01 group being more active than the placebo group (P = 0.006).

### Joint pain score

There were no changes detected within groups over time (D-7 to D28) for either total pain score or index joint pain score. Neither were there any differences between groups at individual time points (data not shown).

### Quality of life (QoL) score

The NV-01 group significantly improved over time (at D28, P = 0.002). The placebo group did not improve over time (at D28, P = 0.376). There were no statistical differences between the groups at any time point for absolute scores, or change in scores. These scores and changes are tabulated in Table 7.

**Table 7:** **Summary of medians, range and statistical comparisons for the Quality of Life index (QoL) at D0 and D28 of the study**

**Group**	**D-7**	**D28**	**D28 minus D-7)**	**Within group change P-value**
NV-01	1730 (20-5940)	500 (0-5520)	677 (-110-2850)	**P = 0.002**
Placebo	2040 (580-9100)	1880 (350-7210)	200 (-1305-4450)	P = 0.376
Between group comparison P-value	P = 0.184	P = 0.060	P = 0.221	

Decreases in scores signify improvement. Significant P-values are denoted in bold type.

### Laboratory values

At screening (Day -7), there were a few notable differences between the groups in hematology parameters, with the placebo group having higher white blood cell count, higher hemoglobin, higher hematocrit and lower mean corpuscular volume. These differences were not statistically significant. Additionally, red blood cell count values were significantly higher in the placebo group (P = 0.024).

At D28, there were several significant differences between the groups in hematology parameters: red blood cell count values were significantly higher in the placebo group (P = 0.004); hemoglobin was significantly higher in the placebo group (P = 0.01); hematocrit was significantly higher in the placebo group (P = 0.011); packed cell volume was higher in the placebo group (P = 0.003).

To further evaluate these findings, these values were examined for significant within group changes. The only significant change within groups was a significant decrease in packed cell volume in the NV-01 group (P = 0.03). No values in any dog were considered clinically significant, and all values remained within the reference range. In the NV-01 group, 9/13 had a drop in PCV, compared to 5/13 in the placebo group. The median values for each group at each time point are documented in Table 8.

**Table 8:** **Summary of the median (min, max) values for each treatment group at screening (D-7) and end of study (D28) for selected hematology parameters**

	**NV-01 D-7**	**NV-01 D28**	**Placebo D-7**	**Placebo D28**
**WBC (x10** ^**3**^ **/UL)**	8.47 (5.34,11.62)	9.27 (5.41,13.22)	9.57 (5.85,14.48)	9.91 (5.72,14.47)
**RBC (x10** ^**6**^ **/UL)**	6.39 (5.38,7.39)	6.06 (5.14,7.25)	6.99 (5.95,8.1)	6.78 (5.69,8.27)
**HGB (G/DL)**	15 (13.1,18.7)	14.65 (11.9,16.7)	16.4 (14.2,18.1)	16.3 (14,19)
**HCT (%)**	44.4 (36.8,55.1)	42.3 (34.8,47.4)	47.2 (42.2,52.2)	47.3 (39.4,53.8)
**PCV (%)**	45 (37,53)	41.5 (35,48)	47 (43,53)	47 (40,53)

WBC = White blood cell count; RBC = red blood cell count; HGB = hemoglobin; HCT = hematocrit; PCV = packed cell volume.

### Anti-NV-01 antibody assay in canine plasma

Using a competition binding ELISA format, the presence of anti-NV-01 neutralising antibody responses was evident if the concentration of NV-01 measured in the presence of plasma from NV-01-treated dogs was markedly lower than the concentration measured in the presence of plasma from placebo-treated dogs or in the absence of plasma. By this assay, no neutralizing antibodies (neutralizing antibodies to the anti-NGF antibody) were detected in the D28 plasma of any dogs tested (n = 12).

## Discussion

Using a randomized, parallel group, stratified, double masked, placebo controlled, proof of principle clinical study design we supported our hypothesis that a single treatment of NV-01 would decrease pain and improve mobility over a 4-week time period in dogs with DJD-associated pain, as measured by owner-completed clinical metrology instruments (CMIs) and objectively measured activity.

These positive findings pertain to dogs where owners have noticed activity or mobility impairment, and in which subsequent diagnostics confirmed the presence of DJD. We believe our findings are transferable to the general canine population suffering from DJD-associated pain. DJD is an umbrella term describing degeneration of synovial and cartilaginous joints [19]. Many studies describe the inclusion of dogs with only OA (or at least do not describe whether dogs also have other DJD) [20,21], however it is our experience [22,23] that dogs with appendicular OA commonly have pain on palpation of regions of the spine, and radiographic evidence of axial skeleton DJD (degenerate intervertebral disks; spinal spondylosis; facet joint OA). Therefore, in order to test NV-01 in a relevant population, we included dogs with appendicular DJD or spinal DJD, or both. All of the dogs in our study had painful appendicular DJD, but in 6 of 26 dogs the index area was in the axial skeleton. All of these dogs had radiographic evidence of facet joint degeneration (OA of the facet joints), but also evidence of intervertebral disk degeneration or spinal spondylosis. It is unknown whether the pain elicited on palpation was a result of facet joint OA or intervertebral disk joint DJD. Regardless, we believe this cohort of dogs reflects the dogs presenting in practice for musculoskeletal pain due to DJD, and we believe the term ‘DJD-associated pain’ more accurately reflects these individuals. We were careful to exclude dogs with any neurological deficits. Unlike the majority of participants in the human clinical studies of anti-NGF mAbs [10-14], we did not target dogs that had failed other treatments, or where previous treatments were not appropriately effective.

Force platforms or pressure-sensitive walkways are often used as an objective measure of changes in limb use in relation to joint pain in dogs [20,22-25]. However, many dogs present with multiple joint OA and only a small subset of the canine population with OA has obvious asymmetry in limb use, which is optimal for kinetic evaluation [26]. We therefore used global measures that pertain to pain relief – owner-completed clinical metrology instruments and an objective measure of activity – rather than focus on measures of limb use.

The CMIs we used have all undergone various degrees of validation (responsiveness, criterion validation), with the CBPI and the LOAD being the most evaluated and considered the most valid [26-29]. The CSOM has not been formally tested for criterion validity, but has shown responsiveness with known analgesics [30] and a putative analgesic [1]. In the present study, all the CMI data led to similar conclusions, with the CSOM and LOAD appearing most sensitive to changes associated with treatment. We found significant positive effects of treatment, and this was surprising given the relatively small numbers of dogs. Recent work [26] found ‘strong’ correlation between the LOAD and the CBPI PSS and PIS. However, it was clear in that study there was not perfect correlation, suggesting that the two CMIs are measuring slightly different aspects and this is supported by our current data. Currently, it is not known exactly how different CMIs vary in what they are assessing. What is clear is that despite attempts to validate the CMIs with objective measures of limb use (ground reaction forces) or activity (accelerometry measures), only weak correlation is found for ground reaction forces (GRF) or accelerometry [26]. One explanation may be that the CMIs are not optimally designed. However, the fact they show robust responsiveness to treatment in masked studies, suggests CMIs, GRFs and accelerometry are measuring different aspects of outcome. This supports the inclusion of these different measures of outcome in clinical studies. An important question that remains unanswered is ‘which of these outcome measures is most relevant to the dog?’ It is becoming increasingly recognized that the different outcome measures are assessing different aspects of the multifactorial pain experience.

Overall, the degree of reduction in pain over placebo across the CMIs was approximately 30% (15-40%), which is considered clinically relevant in human medicine. When evaluating the effect size of the change in LOAD, we found a large effect size at both D14 and D28. Together, our data suggest a relatively large effect of NV-01, but could also represent Type I error. Further studies in larger numbers of dogs are needed to confirm the current findings.

Conversion of CMI data to success/failure has been recently described for the CBPI [31] and the CSOM [30]. We used these recommendations, and found significantly more treatment successes at D28 using the CSOM. This was surprising because to detect significant differences using success/failure analysis requires large differences between the groups or large numbers of animals, and is not usually seen with relatively small group sizes. Additionally, the distribution of success/failure for the CBPI (46%/8%) and CSOM (67%/23%) was very similar to previous studies using the NSAIDs carprofen (CBPI evaluation - 46% success in carprofen group, 23% success in the placebo group [31]) and meloxicam (CSOM evaluation - 73% success in meloxicam group, 47%% success in the placebo group [30]) The present CMI data suggests the efficacy of a single dose of NV-01 is at least identical to that of daily NSAID administration. Currently, the duration of action is unknown, and further work is needed to determine this.

Treatment-related effects on activity as measured by accelerometry were seen, particularly for the day-time period. The accelerometers we used have been shown to be a surrogate measure of dog activity [32], and have successfully detected changes in activity related to NSAID administration in clinical studies in dogs [33]. Activity is highly variable from day to day both within and between dogs [34]. Given this variability, we elected to assess a change in activity over time within individual dogs, using 7-day blocks of time [34]. We detected improved day-time activity in the NV-01 group over the placebo group. Looking at the 24-hour profile of activity at baseline, it might have been expected to see changes in the peaks of activity. However, the peaks of activity (morning and evening) are likely defined by owner activity and owner interaction with the dogs. Conversely, the activity during the daytime (when owners are at work) may be more representative of spontaneous activity. Little work has been performed on how analgesic therapy changes the profile of activity over the day, and further work, including capturing owner schedules, is required to better understand this.

The dose used was based on earlier work with NV-01 that suggested 200 mcg/kg showed analgesic efficacy in the kaolin model of inflammation [16]. Our current data appears to confirm the efficacy of NV-01 and indicates that efficacy lasts for at least 28 days following a single injection. No neutralizing antibodies were detected in any of the dogs treated with NV-01, that is, no antibodies to the anti-NGF antibodies. Further work is needed to determine the pharmacokinetic profile of NV-01 and the duration of efficacy from a single dose. In the human clinical trials, several versions of anti-NGF mAbs have been evaluated, and overall, efficacy is detected within 2 weeks and appears to last at least 8 weeks [10-15]. The majority of patients enrolled into these human clinical trials are patients in which pain was not adequately controlled by NSAIDs and/or opioids, which is in contrast to our canine study inclusion criteria. Of interest, all the basic studies on anti-NGF were performed in rodent models of inflammatory arthritis or inflammatory pain. No studies have been performed in rodent models of OA or non-inflammatory DJD, making this current study the first placebo-controlled evaluation of anti-NGF in a non-human model of non-inflammatory DJD. If further work confirms the efficacy of NV-01 in dogs with DJD-associated pain, it will be a demonstration of the predictive validity of the ‘spontaneously –occurring canine DJD model’ (that is, DJD in owned companion pet dogs) for human conditions.

The present study was not appropriately powered to assess the potential for side effects. In human clinical trials, two main types of side effects have been seen with anti-NGF mAbs – neurological effects and effects on the progression of OA [15]. We performed a neurological evaluation of all dogs at 28 days following treatment and did not detect any neurological abnormalities. Many of the neurological side effects in people were peripheral sensory symptoms, primarily paresthesia, hypoesthesia and hyperesthesia. It is unlikely these would be detected in canine patients by a detailed clinical neurological evaluation such as we performed. Future work should use quantitative sensory testing (QST) methods that are being developed in dogs [35-37] to more completely evaluate sensory function changes. Human clinical studies of anti-NGF mAbs were halted by the FDA due to a signal of adverse events related to rapidly progressing osteoarthritis (RPOA) in approximately 1% of treated patients. This occurred in diseased joints, but there were also some cases that occurred in previously asymptomatic joints [15]. The mechanism of this RPOA is not understood. We saw no cases of worsening joint pain in our study, but a significantly larger cohort of treated dogs would be needed to detect whether or not a similar side effect is seen in dogs. There is no data in veterinary medicine on the normal rate of progression of OA in dogs, and this should be an area targeted with clinical research prior to the advent of anti-NGF mAbs in veterinary medicine. In the present study, we did detect a statistically significant, but clinically benign, change in packed cell volume in the NV-01 treated group. The reason for this is unknown, and it has not been reported in human studies. This should be evaluated carefully in subsequent studies.

## Conclusions

This pilot, masked, placebo-controlled clinical study suggests efficacy of NV-01, a monoclonal canine-specific antibody against nerve growth factor, in reducing spontaneous DJD-associated pain and improving mobility in dogs. Owner-assessed indices of pain and mobility were significantly improved in NV-01 treated dogs compared to placebo, and objectively measured activity increased significantly more in the NV-01 treated dogs during the daytime period.

## Methods

### Design

This was a randomized, stratified, double masked, placebo controlled, proof of principle clinical pilot study with parallel treatment groups. The Institutional Animal Care and Use Committee (IACUC) approved this study (IACUC #12-149-O), and in all cases owners signed a written consent form following a detailed verbal explanation of the study protocol.

### Study population

Dogs ≥1-year old and ≥15 kg with DJD-associated pain and mobility impairment were recruited. Recruitment was performed using advertisements on the NCSU-CVM internal TV monitors, through NCSU websites, e-mail advertisement to NC State university colleges (Education, Engineering), via direct advertising to local veterinarians and newspaper advertisements run in local weekly newspapers. Promotion of the study was also done using the NCSU-CVM social media sites (NCSU-CVM blog, Twitter feed and Facebook page). Recruitment began mid-April 2013 and continued through mid-October 2013.

### Inclusion criteria

To be eligible for the study, dogs were required to have impaired mobility of a certain severity, as judged by owners, no detectable systemic disease, and at least one appendicular joint or axial skeleton area that was considered painful and where radiographs showed the presence of DJD (see ‘Study Protocol’). Radiological features used to establish the presence of DJD in appendicular joints were: joint effusion, osteophytes, sclerosis, subluxation, subchondral bone erosions and cysts and presence of intra-articular mineralization. Radiological features used to establish the presence of DJD in the axial skeleton were: osteophytes, spondylosis, disc-associated degeneration (end plate sclerosis, erosion, disc mineralization, narrowing) and subluxation. Additionally, dogs were required to not be currently receiving any anti-inflammatory medications (NSAIDs) or other analgesics (e.g. amantadine, gabapentin, tramadol). Other analgesics (e.g. amantadine, gabapentin, tramadol) were permitted if it was deemed there was currently significant pain associated with the DJD, and the dog had been on the medication(s) for at least 3 weeks. Dogs were required to be either not receiving nutritional supplements, or have been on them for 6 weeks or more before the start of the study. A two week withdrawal period was required prior to study entry for dogs discontinuing nutritional supplements, NSAIDs or other analgesics. If dogs were considered to be mobility impaired but no DJD was detected radiographically, or if they had DJD but the impairment in mobility was not sufficient, they were not enrolled. Other exclusion criteria included known or suspected presence of any of the following conditions: clinically significant cardiovascular disease; severe dental disease; neurological disease, renal disease; liver disease (ALT levels of up to twice the upper normal value and AlkPhos levels of up to four times the upper normal value were considered acceptable in the absence of other signs of liver disease); chronic pulmonary disease; infectious disease; immune-mediated disease; neoplasia; urinary tract infection; hypothyroidism (unless well controlled); diabetes mellitus; skin disease of the foot; obesity (8 or 9 out of the 1-9 Body Condition Score Scale). These were exclusion criteria because they may be associated with decreased activity that would not respond to analgesic treatment. Particular attention was given to ruling out neurological disease through a comprehensive neurological evaluation. Additionally, owners had to agree to not change the management of dogs for the period of the study, and owners were required to have a stable lifestyle for the duration of the study (no planned house moves, vacations, relationship changes or new pets).

### Study protocol

The study was conducted over a 28-day period with outcome measures gathered at screening (D -7) and on D0, D14 and D28.

Approximately 7 days prior to starting on the study, potential candidate dogs were screened using a general physical, neurological and orthopedic examination, complete blood count, serum biochemistry and urinalysis. Physical and orthopedic examinations were performed by the same examiner (MF) throughout the study. Pain on manipulation of each appendicular joint and manipulation of each region of the axial skeleton was assessed and recorded using a 5-point scale (Additional file 1). Scores were summed across all appendicular joints (manus, carpus, elbow, shoulder, pes, hock, stifle, hip) and each region of the axial skeleton (cervical, thoracic, thoraco-lumbar, lumbar, lumbo-sacral) to create a total pain score (maximum score 84). In addition, an index joint or region of the axial skeleton was defined, and the index joint/region pain score recorded. The index joint/region was defined based on clinical judgment of what was the most severely affected area, with the predominant criterion being the pain score for that area. Based on the orthopedic examination (pain and function), dogs were designated as being either predominantly ‘Fore’ (forelimb, neck or thoracic spine) or ‘Hind’ (hind limb, thoraco-lumbar, lumbar or lumbo-sacral spine) impaired. Digital radiographs of all clinically abnormal (painful) appendicular joints or areas of the axial skeleton were taken under sedation. Owners completed three clinical metrology instruments (CMIs) - the Client Specific Outcome Measures (CSOM) [1,30] the Canine Brief Pain Inventory (CBPI) [27,28] and the Liverpool Osteoarthritis in Dogs Index (LOAD) [26,29]. Owners also completed a Quality of Life Index (Additional file 2), constructed in a similar manner to previous reports [38,39]. Dogs were required to have a qualifying degree of mobility impairment as reported and scored by the owner on the CSOM (CSOM score of ≥5, based on three activities each scored on a 0-4 scale where 0 = normal, 4 = impossible to perform the activity). Dogs were fitted with a collar-mounted accelerometer (see below). Owners were asked to keep a diary of any unusual events that might affect a dog’s activity.

On D0, owners returned to the clinic with their dogs, and completed the CMIs again. The physical, orthopedic and neurological examinations were repeated to ensure there were no changes and no additional findings. On D0, dogs were administered the anti-nerve growth factor antibody (NV-01) or placebo, at a dose of 200 mcg/kg of a 2 mg/ml solution administered IV over a 1-minute period through an intra-venous 20G catheter. Normal saline was used as the placebo, and administered IV at a volume equivalent to the dose of NV-01. Following test article administration, dogs were observed for a period of 4 hours for any signs of an allergic reaction.

On D14, owners returned to the clinic and completed the CMIs and the physical, orthopedic and neurological examinations were repeated to ensure there were no changes or additional findings.

On D28, owners returned to the clinic and completed the CMIs, the physical, orthopedic and neurological examinations were repeated and joint pain scored. The data from the accelerometer was downloaded, and samples collected for CBC, clinical chemistry and urinalysis.

### Randomization to treatment groups

Dogs were randomized to receive the drug or placebo on D0 based on a stratified, blocked design. Stratification was based on predominant site of problem (Fore or Hind), and total CBPI score (<50, ≥ 50). Hence, the stratification groups were: CBPI < 50/Fore; CBPI < 50/Hind; CBPI ≥ 50/Fore; CBPI ≥ 50/Hind.

Within each stratification, randomization of treatment occurred in groups of 2 (e.g. placebo, then NV-01; or, NV-01 then placebo). The randomization schedule and the key were held by the NCSU pharmacy and not disclosed to investigators. The dispensing of drug/placebo was performed by NCSU pharmacy, with all other personnel involved in the evaluation of dogs and collection of data masked to the administration until completion of the statistical analysis. Pharmacy staff prepared unmarked syringes for each patient with the barrel covered in opaque tape. Testing prior to starting the study indicated there was no appreciable difference between the feel of injecting saline versus NV-01 through a 20G catheter, allowing masking to be complete.

### Outcome measures

The primary outcome measures were the CMIs and actimetry data. Secondary outcome measures were index joint pain, total joint pain score, owner-assessed side effects, hematology, clinical chemistry and urinalysis.

#### Clinical metrology instruments

CMIs were completed by the same owner at all visits, and owners were directed to base their answers on their observations of the preceding seven days. The owners completed the CMIs while sitting or standing (as they preferred), in a standard consulting room. At each time point, they were requested to complete the CMIs and the instructions were explained to them in a neutral tone of voice. Owners completed the CMIs while their dog was being examined in a separate room.

The LOAD [26,29] is a 13-item instrument with all items reported on a five-point Likert-type scale. Each item is scored 0 to 4, and the item scores are summed to give an overall instrument score. The CBPI [27,28] is a two-part instrument. The pain severity score (CBPI PSS) is the arithmetic mean of four items scored on an 11-point (0 to 10) numerical scale, and the pain interference score (CBPI PIS) is the mean of six items similarly scored. The CSOM [30] is a CMI that follows 3 activities unique to the individual dog that are determined to be impaired. It is modeled after the Cincinnati Orthopedic Disability Index, CODI [40]. The CSOM was constructed by a single study individual (BC) for each case as previously described [1] but only defining 3 activities as more recently described [30]. At each time point, the difficulty performing each of the 3 activities was scored on a 0-4 scale: 0 = No Problem, 1 = Mildly Problematic, 2 = Moderately Problematic, 3 = Severely Problematic, and 4 = Impossible. The CSOM score resulted from the addition of the score for each activity. Only complete CMIs were considered valid.

#### Activity monitoring (AM)

As previously reported [23,26,33], the spontaneous activity of each individual dog was measured using an accelerometer^a^, a method termed actimetry. Actimetry commenced following screening and continued for the duration of the study. At D28 the monitor was removed from the collar and placed on a telemetric reader to download the data to a personal computer.

### Statistical analysis

This was a pilot study, and the potential degree of improvement associated with anti-NGF antibody was not known, and so the number of dogs was based on the following. From a previous study carried out at our site [23], change in mean pain interference CBPI score following 2-weeks of an NSAID was 1.4 (baseline = 2.9; after NSAID for 14d = 1.5) and the mean standard deviation was 1.2. Using this standard deviation, and data from a study [28] where the improvement in CBPI interference score was 0.2 for placebo, and 1.1 for NSAID, we determined that a total of 24 dogs (12 each group), would give a power of 0.49 which was considered acceptable for a pilot study.

All data were entered into hard-copy notebooks, and then transcribed to digital files. All data underwent 100% quality control by two individuals not associated with the study to verify the digital entries. Descriptive statistics were used to describe the demographic characteristics of the two groups, and appropriate statistical tests used to compare these characteristics.

For all subjective parameters (CMIs, joint pain, QoL), the change within groups over time, and the difference in change between groups were assessed statistically. Data were compared non-parametrically. Data were converted to success/failure where published guidelines were available, and analyzed using a Fisher’s Exact test. For the CBPI data, success/failure at D14 and D28 was defined based on an improvement of at least 1 for CBPI PSS, and at least 2 for CBPI PIS, with inclusion criteria set as a CBPI PSS and CBPI PIS of at least 2 at baseline (D0). For the CSOM, success/failure at D14 and D28 was defined as an improvement of 2 or more on the total CSOM, with no deterioration in any single CSOM activity.

Actimetry data were extracted for days -7 to -1 (baseline), and 20 to 27 (final week of study). For each dog, the average activity count per minute over each 7-day period was calculated and used for analysis. Activity data were normally distributed, and within-group changes evaluated using a paired *t*-test. Between group comparisons were not performed due to the large inter-dog variations in baseline activity, and recent data from our group indicating significant differences in output between accelerometers (unpublished data). In addition, the within-group change in mean activity per minute for each hour of the day was compared for several segments of the day.

Laboratory data were compared between groups at the start and end of the study (Wilcoxon Rank Sums). Where significant differences between groups were detected, they were further examined for within group changes (Wilcoxon Signed Rank).

In all analyses, the critical P-value was set as 0.05. Due to the pilot nature of the study, the P-value was not adjusted for multiple comparisons within any outcome measure, and additionally, in some results, both the one-sided and two-sided statistical test results are described.

### ELISA assay for neutralizing antibodies to NV-01

Plasma samples were assayed in a competition ELISA for inhibition of binding of purified NV-01 to mouse NGF. Purified NV-01 was mixed with dog plasma from placebo- or NV-01-treated dogs and added to plates previously coated with mouse NGF. Following incubation, blocking and washing, binding was detected using secondary anti-canine IgG polyclonal antibody-HRP conjugate [16]. Purified NV-01 without dog plasma, in the absence or presence of a neutralizing anti-NV-01 mouse monoclonal antibody (1RC1) was used as negative and positive controls.

## Endnote

^a^Actical Activity Monitor, Philips Respironics Co, Bend, OR.

## Additional files

Additional file 1:
**Orthopedic examination pain scale.**
Additional file 2:
**Global weighted quality of life score (canine).**

## Abbreviations

AE: Adverse event
AlkPhos: Alkaline phosphatase
ALT: Alanine transferase
CBPI: Canine brief pain inventory
CCL: Cranial cruciate ligament
CSOM: Client specific outcome measures
CVM: College of Veterinary Medicine
DJD: Degenerative joint disease
ELISA: Enzyme-linked immunosorbent assay
FDA-CVM: Food and Drug Administration – Center for Veterinary Medicine
GRF: Ground reaction forces
HCT: Hematocrit
HGB: Hemoglobin concentration
LOAD: Liverpool osteoarthritis in dogs index
mAb: Monoclonal antibodies
NCSU: North Carolina State University
NCSU-CVM: North Carolina State University, College of Veterinary Medicine
CPRL: Comparative Pain Research Laboratory
NSAIDs: Non-steroidal anti-inflammatory drugs
OA: Osteoarthritis
PCV: Packed cell volume
PSW: Pressure sensitive walkway
QoL: Quality of life
QST: Quantitative sensory threshold
RBC: Red blood cell count
RPOA: Rapidly progressing osteoarthritis
WBC: White blood cell count
