# Supplementary material for: A canine-specific anti-nerve growth factor antibody alleviates pain and improves mobility and function in dogs with degenerative joint disease-associated pain
Source: BMC Vet Res. 2015 Apr 30;11:101. doi: 10.1186/s12917-015-0413-x (PMC4419463; doi:10.1186/s12917-015-0413-x)
Supplement: Additional file 1: — Orthopedic examination pain scale. [file 12917_2015_413_MOESM1_ESM.docx]

Additional file 1

Orthopedic examination pain scale

Manipulate the joint, or palpate and manipulate the axial skeleton by repeated and gradually increasing pressure associated with palpation, and also by moving the area through a normal range of motion. Following this, score the patient as per below. Use whole numbers given.

0 Does not notice manipulation

1 Orients to site on manipulation, does not resist

2 Orients to site, slight objection to manipulation

3 Withdraws from manipulation, may vocalize, may turn to guard area

4 Tries to escape from manipulation, or prevent manipulation, may bite or show aggression on manipulation
