# Supplementary material for: A canine-specific anti-nerve growth factor antibody alleviates pain and improves mobility and function in dogs with degenerative joint disease-associated pain
Source: BMC Vet Res. 2015 Apr 30;11:101. doi: 10.1186/s12917-015-0413-x (PMC4419463; doi:10.1186/s12917-015-0413-x)
Supplement: Additional file 2: — Global weighted quality of life score (canine). [file 12917_2015_413_MOESM2_ESM.docx]

Additional file 2

Global weighted quality of life score (canine)

What activities or things your dog does are most important for your dog’s quality of life (as far as you are concerned)?

| ACTIVITY | IMPORTANCE SCORE (OUT OF 100: note, the 5 scores must add up to 100) |
| --- | --- |
| Activity 1 |  |
| Activity 2 |  |
| Activity 3 |  |
| Activity 4 |  |
| Activity 5 |  |

Please rate your dog’s current ability to perform this activity:

|  | VAS-Visual Analog Scale |
| --- | --- |
| Activity 1 | Normal Impossible |
| Activity 2 | Normal Impossible |
| Activity 3 | Normal Impossible |
| Activity 4 | Normal Impossible |
| Activity 5 | Normal Impossible |

Legend: Owners were asked to write down five activities (items) that they believed were important for their dog’s quality of life / enjoyment of life. Secondly, they were asked to rate the importance of each item, with the total importance score (IS) across the five items adding up to 100. Following collection of the IS owners were asked to rate their dog’s ability to perform each of the activities listed using a visual analogue scale (VAS) system, marking on the line between ‘normal’ and ‘impossible’ to describe their dog’s ability to perform each activity. Normal on the VAS was designated 100, and impossible was designated 0. The overall Quality of Life Score (QoLS) was calculated as the sum of the scores obtained after multiplying each importance score (IS) by the VAS-ability score for each activity, (QoLS = Σ (ISxVAS)).
